# Saccades during visual exploration align hippocampal 3–8 Hz rhythms in human and non-human primates

**DOI:** 10.3389/fnsys.2013.00043

**Published:** 2013-08-30

**Authors:** Kari L. Hoffman, Michelle C. Dragan, Timothy K. Leonard, Cristiano Micheli, Rodrigo Montefusco-Siegmund, Taufik A. Valiante

**Affiliations:** ^1^Department of Psychology, Centre for Vision Research, York UniversityToronto, ON, Canada; ^2^Department of Biology, Centre for Vision Research, York UniversityToronto, ON, Canada; ^3^Neuroscience Graduate Diploma Program, York UniversityToronto, ON, Canada; ^4^Division of Fundamental Neurobiology, Toronto Western Hospital Research InstituteToronto, ON, Canada; ^5^Krembil Neuroscience CenterToronto, ON, Canada; ^6^Division of Neurosurgery, Department of Surgery, University of TorontoToronto, ON, Canada

**Keywords:** theta, electrocorticography, epilepsy, saccades, phase-locking, macaque, human, foraging

## Abstract

Visual exploration in primates depends on saccadic eye movements (SEMs) that cause alternations of neural suppression and enhancement. This modulation extends beyond retinotopic areas, and is thought to facilitate perception; yet saccades may also influence brain regions critical for forming memories of these exploratory episodes. The hippocampus, for example, shows oscillatory activity that is generally associated with encoding of information. Whether or how hippocampal oscillations are influenced by eye movements is unknown. We recorded the neural activity in the human and macaque hippocampus during visual scene search. Across species, SEMs were associated with a time-limited alignment of a low-frequency (3–8 Hz) rhythm. The phase alignment depended on the task and not only on eye movements *per se*, and the frequency band was not a direct consequence of saccade rate. Hippocampal theta-frequency oscillations are produced by other mammals during repetitive exploratory behaviors, including whisking, sniffing, echolocation, and locomotion. The present results may reflect a similar yet distinct primate homologue supporting active perception during exploration.

## Introduction

For most primates, exploration of the environment is primarily visual, and makes use of the specialized mechanism of saccadic eye movements (SEMs): the rapid and repetitive displacement of a high-acuity region of the retina to sample different locations in the visual environment. During SEMs, neural activity is suppressed (Latour, 1962; Burr et al., 1994; Reppas et al., 2002; Thiele et al., 2002; Uematsu et al., 2013) whereas after SEMs, during fixation, neural activity is enhanced (Ibbotson et al., 2007, 2008; Cloherty et al., 2010). This fluctuation is thought to promote efficient processing of new visual information (Melloni et al., 2009; Schroeder et al., 2010).

Although saccades are known to modulate visual perception, the most notable effects of saccadic activity have been observed in early and intermediate visual areas (Bair and O'keefe, 1998; Leopold and Logothetis, 1998; Martinez-Conde et al., 2000; Reppas et al., 2002; Thiele et al., 2002; Ibbotson et al., 2007, 2008; Maldonado et al., 2008; Rajkai et al., 2008; Bremmer et al., 2009; Crowder et al., 2009; Cloherty et al., 2010; Ibbotson and Krekelberg, 2011; Ito et al., 2011). In the temporal lobe, eye movements made in the dark or following simple visual stimuli elicited spiking and/or local field potential (LFP) modulation in subdomains such as the superior temporal polysensory area, the parahippocampal gyrus, inferotemporal cortex, and hippocampus, though the consequences for perception are unclear (Ringo et al., 1994; Sobotka et al., 1997, 2002; Purpura et al., 2003; Bartlett et al., 2011; Jutras et al., 2013).

The interaction between neural responses to saccades and to visual stimulation illustrates one role of eye movements in active vision. Eye movements that are concomitant with the onset of new visual information, as occurs during naturalistic visual search, lead to temporal lobe neuronal activity which is phase-locked in the theta-alpha range (Bartlett et al., 2011). This coupling effect is stronger than that predicted exclusively by the visually-evoked response when decoupled from fixation, and is generally consistent with reports of phase-locking and phase-dependent codes in early visual areas (Montemurro et al., 2008; Rajkai et al., 2008; Bosman et al., 2009; Ito et al., 2011). Precisely-timed responses through neural synchronization may facilitate the speed and efficacy of perceptual processing (Masquelier et al., 2009; Panzeri et al., 2010; Turesson et al., 2012; Womelsdorf et al., 2012; Lisman and Jensen, 2013), but such mechanisms are not limited to perception. Precise phase-alignment of spiking activity to oscillations in the rodent hippocampus is posited to be relevant for memory encoding, as well (Tort et al., 2009; Shirvalkar et al., 2010; Lisman and Jensen, 2013).

The hippocampus produces low-frequency theta oscillations (5–10 Hz) during whisking, sniffing, and locomotion in rodents (Grastyán et al., 1959; Vanderwolf, 1969), during echolocation in the bat (Ulanovsky and Moss, 2007), and during passive viewing of images in macaques (Jutras et al., 2013, but see Skaggs et al., 2007). These oscillations are, in turn, associated with encoding of experiences for later recall, for review see Buzsáki (2005); Hasselmo (2005). The role of eye movements in modulating hippocampal oscillations during active search is unknown. Here we asked whether saccades influence hippocampal oscillations during a visual foraging task measured in the human and non-human primate.

## Materials and methods

### Participants

Six patients (3 males) with medically refractory epilepsy underwent surgical implantation of subdural surface electrodes and depth macroelectrodes to localize epileptogenic regions. Electrode location and type were selected solely on clinical considerations. These experiments occurred between 2 and 10 days post-operatively, with informed consent, and in accordance with protocols approved by the University Health Network Research Ethics Board and the York University Human Participants Review subcommittee. A single adult rhesus macaque (*Macaca mulatta*, female, 10 kg) also participated in these experiments following implantation of chronically indwelling tetrodes. The experiments occurred 2–5 months post-operatively, under protocols approved by the Animal Care Committee at York University, in accordance with the Canadian Council for Animal Care.

### Experimental design

The basic experimental design is described previously (Chau et al., 2011). Exceptions are noted in the summary below.

#### Stimuli

Photographic images were taken from a large collection of natural scenes including landscapes, cityscapes, wildlife, and indoor scenes. One object in a given image was modified in Adobe Photoshop to give the impression that it disappeared, referred to here as the “target.” Sizes, locations, and content of targets were varied to reduce the predictability of the target object by prior experience with the task. Images were presented full-screen at 1280 × 1024 pixel resolution.

#### Behavioral procedure

Each participant sat approximately 51 cm away from a 38-cm by 30-cm computer monitor. Patients sat in a small testing room under fluorescent lighting with 2–3 experimenters in the room with them. Macaques sat in a private darkened booth inside a room where the experimenters monitored the neural and behavioral activity. All participants first underwent a 13-point calibration of the eye tracker system (patients: iView RED sampled at 60 Hz: macaque: iView high-speed primate, sampled at 1250 Hz, both from SensoMotoric Instruments, Teltow, Germany). The eye tracker was connected via ethernet cable to the stimulus presentation computer running Presentation (NeuroBehavioral Systems, Albany, CA, USA). During each trial of the main task, an original scene was shown in alternation with the target-modified scene, each lasting 500 ms, with an intervening 50-ms gray screen separating these image pairs. Participants searched for the single changing object—the target—and, upon detection, could elicit the end of the trial by fixating the target for 1000 ms. Regardless of whether or not the target was found within the time limit (typically 45 s), the trial ended by removing the gray screen gap, revealing the target as the only changing part of the image thereby removing the “change blindness” illusion. In the case of the macaque, if the target was found in the time limit, this target “giveaway” was accompanied by delivery of a preferred smoothie treat. All participants of the main task viewed trials in blocks of 30, with the number of blocks per analysis varying from 4–10 in the patient participants and spanning 37 blocks for the macaque. With equal probability, the scene pairs in a given trial were either novel or repeated once from a previous trial, and targets were unique to the scenes, i.e., uncued. All but one participant completed this main task.

The remaining participant, Patient 6, was run on a control variation of the task in which only one image of the pair was presented, and with a presentation duration of up to 6 s, after which the target location was revealed through alternation, as described above. Two successions of 10 unique targets-in-scenes were presented, and each succession was repeated 3 times, for a total of 80 trials. As with the main task, the trial ended before the time limit if the target location was fixated for 1000 ms. This control task provided a constant image during exploratory saccades, similar to other memory-guided search tasks (Ryan and Cohen, 2004; Smith et al., 2006; Chau et al., 2011; Chukoskie et al., 2013), removing the interposition of image onsets with saccades, as occurs during the main task.

The inter-trial interval procedures differed across patient and task. In the main task, patients saw a series of screens prompting verbal responses for memory of the scenes and target objects. The macaque was not asked for verbal report but was instead given a 20-s inter-trial interval in which the display was set to black. Similarly, in the control task (Patient 6) the end of the trial proceeded directly to a black display screen lasting 5 s, with no verbal report screen.

### Neural recordings

Electrophysiological recordings in patients were obtained from depth macro electrodes with four electrical contacts used to record hippocampal activity. In addition, patients were implanted with strips of 4–6 subdural platinum-iridium electrodes 3-mm diameter and 10-mm inter-electrode distance (PMT, Chanhassen, MN, USA) targeting anterior temporal, ventral-medial temporal, and posterior temporal locations. A 4-contact subgaleal electrode over the parietal midline and facing away from the brain was used for ground and reference. Signals were filtered at 0.1 Hz–1 kHz, sampled at 5 kHz with a NeuroScan SynAmps2 data acquisition system (Compumedics, Charlotte, NC, USA), and recorded to disk. Electrode localization was verified by co-registering a post-operative CT image with a pre-operative MRI structural image.

In the macaque, quartz platinum tungsten tetrodes (Thomas Recordings, Giessen, Germany) were implanted chronically in a modified 18-drive (Neuralynx Inc, Gray Matter Research, Bozeman, Montana, USA). The guide tube was insulated until the tip, which ended ~4 mm above the hippocampus and served as a local reference. Each tetrode was independently adjustable in depth up to 1 cm. Signal was split between spiking and LFP channels digitally and sampled at 32 and 2 kHz, respectively, using a Digital Lynx acquisition system (Neuralynx, Inc.). LFP was filtered between 0.5 Hz and 2 kHz. Electrode location was determined functionally with characteristic hippocampal activity and structurally using post-operative MRI.

For both neural acquisition systems, serial-output pulses from the Presentation stimulus-delivery PC were used to synchronize neural and behavioral events.

### Data analysis

Eye tracking files were preprocessed with iView X iTools IDF Event Detector, using a dispersion based algorithm (I-VT) with a minimum fixation duration of 80 ms and maximum dispersion of 100 pixels (Salvucci and Goldberg, 2000).

Neural and eye movement data files were read into MATLAB (The Mathworks Inc., Natick, MA), and processed with purpose-built code and the FieldTrip toolbox (Oostenveld et al., 2011). LFP signal preprocessing included resampling to 1 kHz, filtering between 1 and 200 Hz, detrending and—for patient data but not macaque data—a notch filter was applied in preprocessing at 30 and 60 Hz to remove line noise and artifacts. Neural data underwent artifact rejection through the FieldTrip toolbox. No interictal spikes were observed to occur in the data presented here. Subsequent to testing in this experiment, some electrodes were identified as recording from epileptogenic regions (see Table 1, Figure 4D).

**Table 1:** **Subject and electrode recording details**.

**ID**	**Sex**	**Seizures**	**LH**	**RH**	**LTP**	**RTP**	**Saccades**	**Per trial**	**Peak (Hz)**
P1	F	L Hipp	1	2	2	3	5287	36.4	7.5
P2	F	L Hipp	2	0	1	0	9795	44.3	8.0
P3	M	L+R Hipp	1	1	1	2	6960	38.4	8.3
P4	M	R TL	1	1	1	1	8388	32.6	9.5
P5	M	L Hipp	2	0	2	0	6970	33.2	10.3
P6^*^	F	OFC	0	1	N/A	N/A	1118	10.2	7.1
MM1	F	N/A	0	4	0	0	10–27k	56.7	8.2

For each participant, the sex, epileptogenic zone, number of recordings sites for a given location, number of saccades, median number of saccades per trial, and fast Fourier transform (FFT) trial-averaged theta peak is shown. The number of saccades for M1 varied by recording site (var). Abbreviations are: P, patient; MM, macaca mulatta, F, female; M, male; L, left; R, right; Hipp, hippocampus; OFC, orbitofrontal cortex; TL, temporal lobe neocortex; TP, temporal pole. Underlined numbers indicate post-experiment seizure activity in that hippocampus.

* Patient 6 ran a control task; data were analyzed separately, see Materials and Methods.

Exclusion criteria for eye movements were: 1. any eye movement occurring within the first 1 s of the trial, when strong image-evoked activity occurs and to allow pre-and post- fixation windows to reflect similar visual stimulation conditions, i.e., after image onset. 2. For trials in which the target was found before the time limit, we excluded fixations in the target area of interest that led to the “target found” trigger. In this way we are isolating eye movements during search and not as part of target fixation and recognition. Eye movements were also collected in the inter-trial intervals of the macaque experiment, while in the darkened booth, and in the control task with Patient 6, on the black screen in the lit room. For the analysis of long fixations, we took the subset of fixations that lasted >500 ms and that were immediately preceded by a fixation whose duration was also >500 ms, thereby creating an analysis window that was not contaminated by additional eye movements, and that reflected an eye movement rate of <2 Hz, i.e., below the neural frequencies of interest.

In general, statistical significance was determined in two steps. First, a null distribution was created by shuffling the saccade times in each trial, for all trials for that electrode site, calculating the analytical measure of interest for each data point, and repeating this process 1000 times to identify observed values that exceeded the 0.001 threshold of the null distribution for that data point. Second, a Benjamini–Hochberg FDR correction was applied to the time or time-frequency series of interest, to correct for multiple comparisons. Specific details or exceptions are noted below. The within-trial shuffling was selected to allow an identical number of samples per trial, and trials per analysis, and the same across-trial variability as the original data, jittering only the precise timing to the onset of a given fixation.

Fixation-aligned mean evoked responses (Figure 2) were considered significant if the mean observed response exceeded the 0.002 percentile of the mean shuffled-fixation response distribution (a two-sided test of the 1000-element null distribution). Time-frequency plots aligned to fixation onset were calculated with FieldTrip using a Hanning window of 800 ms from −1.2 to 1.2 s (i.e., windows centered from −800 to 800 ms) taken every 10 ms, in 1-Hz increments from 3 to 80 Hz (Figures 3, 4) and from 3 to 20 Hz (Figure 5). The Hann (or Hanning) window was selected rather than multiple tapers to maximize temporal precision with minimal spectral leakage for these time-limited, low-frequency events of interest. Peri-fixational changes in power were tested by comparing an observed time-frequency power value to those of its fixation-time shuffled distribution, and FDR-correcting for the number of time-frequency points tested.

Phase analysis used the same time and frequency windows as the power analysis. Phase alignment was calculated as the pairwise phase consistency, “PPC,” (Vinck et al., 2010). Briefly, the PPC—like other measures such as the phase locking value (PLV)—is a measure of the consistency of phase across observations for a given frequency. Whereas PLV calculates phase for each event (here, fixation), and then determine the central tendency of the distribution, the PPC calculates the circular distance between pairs observations (the cosine of the absolute angular distances), permuted over the population of events, from which central tendency is then measured. The reasoning is that if events are aligned to a common phase, the average absolute angular distance among event pairs (i.e., among relative phases) will be small. We also calculated the PLV measure for comparison and, as previously established analytically, the PPC approximates the square of the PLV.

For the results shown in Figures 4, 5, observed PPC values were compared to a permuted distribution containing the PPC values obtained when fixation onset times shuffled within-trials (over 1000 permutations). Significance masks were set at the 0.001 threshold of this permuted distribution and also had to survive multiple comparison correction using the Benjamini–Hochberg FDR correction of the Rayleigh distribution, set to *q* < 0.001. To test the frequency specificity of peri-fixational phase alignment, windows centered from −50 to 250 ms from fixation onset were selected for each frequency 3–80 Hz and tested against the fixation-time shuffled distribution at the respective frequency. Frequencies with PPCs exceeding the *p* < 0.001 threshold and also surviving the FDR correction are indicated in Figure 4C. Temporal selectivity was tested by comparing these peri-fixational PPC values to those obtained from a window centered −800 to −600 ms prior to fixation onset, i.e., “pre,” and measured using a Wilcoxon signed rank test “signrank” (Figure 4D). PPC data from each individual hippocampus was tested for values exceeding *p* < 0.001 of its fixation-shuffled distribution obtained over the same time window. The individual results are presented in Figure 4E as a normalized index: (PPC_peri - PPC_pre)/(PPC_peri + PPC_pre), allowing an appreciation of the degree of fixation-related phase alignment above that seen prior to the fixation.

Figure 5 shows the PPC in restricted frequency bands across control conditions. As before, each PPC value is compared to its fixation-shuffled distribution and FDR corrected for its respective plot. Only values exceeding both thresholds survive the mask. For 5A, the original and a subset of data are shown; the subset includes only long fixations before and after the fixation event of interest.

## Results

### Search behavior

**Figure 1:**
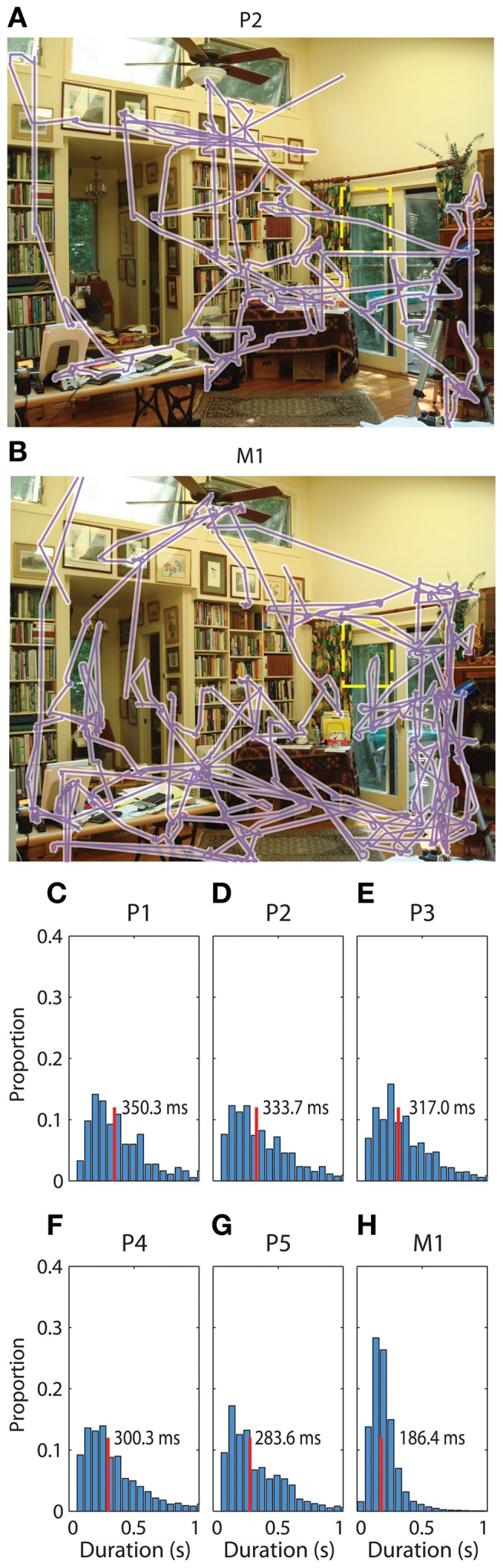
**Saccadic eye movements during visual search. (A)** Gaze during one example trial from patient 2. The sequence of gaze locations is shown in purple. The target from this trial was a wind chime, which has been outlined with a yellow dashed rectangle for visibility in this figure. The patient spent the full 45 s searching for the target. **(B)** Gaze during the same trial viewed by a macaque. Conventions are as in **(A)**, and the macaque also did not find the target on this trial. **(C–H)** Normalized histogram of fixation durations during the search task for each of the six participants in the main task, respectively. Median search times are listed and indicated with a vertical red line.

The seven participants in this study contributed between 1000 and 28000 fixation events during visual scene search (Table 1). All participants actively scanned the image in search of the “target” object (Figures 1A,B for examples) with median fixation durations between 180 and 350 ms across participants (Figures 1C–H).

### Recording locations

**Figure 2:**
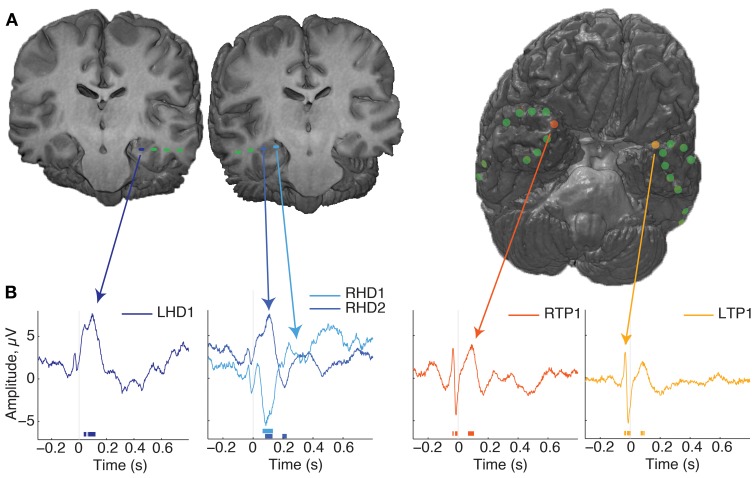
**Localization of electrodes and evoked responses. (A)** Renderings from patient 1's MR images with co-localized recording sites. On the left are two coronal sections showing the location of hippocampal depth macroelectrodes, with the most eccentric 1–2 contacts localized to the hippocampus, indicated by the blue dashes. On the right is a rendering of the whole brain, with the anterior tip oriented up and to the right, revealing the locations of subdural surface electrodes. This was the most common arrangement for the patients in this study. **(B)** Average evoked responses from several electrode locations, aligned to fixation onset. Time is on the x-axis, relative to fixation onset and magnitude in microvolts is on the Y axis. Time points of significant deviations are indicated by the lines at the bottom of the plot, color coded for the corresponding site (*p* < 0.001 cutoff of the fixation-time shuffled distribution). Note that the post-fixation response is qualitatively strongest in the hippocampus, and shows a polarity reversal across RHD sites; other locations such as the anterior temporal lobe show transient, broad-band modulation around the saccade event. For more information of the electrode locations sampled across subjects, see Table 1.

We determined patient recording locations from CT co-registration to the pre-operative MR independently from electrophysiological signal analysis. In addition, the macaque recordings included functional characteristics of hippocampal activity such as sharp wave ripples, and complex spikes, as defined in previous studies (Skaggs et al., 2007). Not all surface electrodes in patients sampled the same regions, with the exception of co-localized temporal pole sampling (e.g., Figure 2A, Table 1).

### Evoked responses

Recordings aligned to fixation onset revealed several patterns of modulation. A fast, transient response just prior to fixation was seen at most sites (green lines, Figure 2B, reflect this across-electrode common response). In comparison to the average, the pre-fixation transient was strongest at the temporal pole sites. The hippocampal probes showed a response following fixation not common to other sites, and which could manifest itself across multiple HC contacts, as opposite-polarity signals, depending on hippocampal recordings site (Figure 2B, second plot). Slight fluctuations in frequency and phase of responses across trials can lead to attenuation of the evoked response, so higher frequencies and sustained responses can be difficult to measure. To evaluate the relative strength of oscillations independent of polarity and of evoked oscillations associated with fixations, we calculated the trial-by-trial spectral power.

### Spectral power

**Figure 3:**
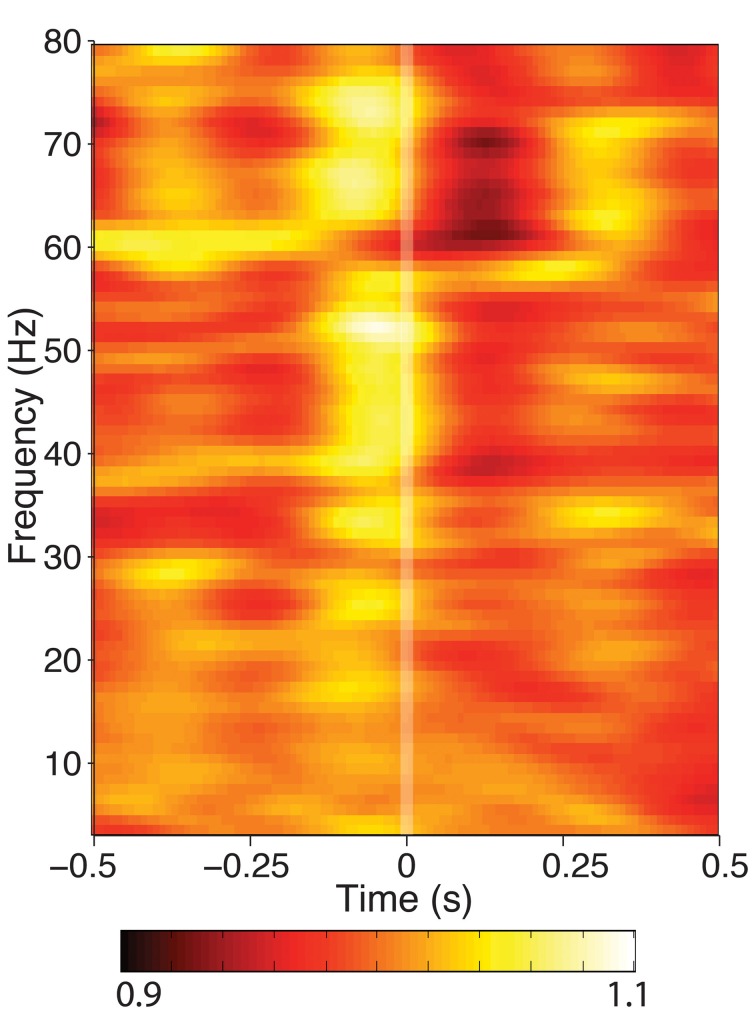
**Time-frequency spectrogram of hippocampal activity aligned to fixation onset during visual search, grand averaged across each patient**. Frequency was measured from 3 to 80 Hz at 1 Hz intervals, time was measured in 800 ms Hanning-tapered windows, shifted every 10 ms. For visualization of each frequency band, power is presented in each band relative to the average seen for that frequency band between ±800 ms around fixation onset. None of the hippocampal recording sites showed theta power modulation, though some exhibited modulation in higher frequency bands.

When mean spectral power was calculated from segments of data aligned to fixation during search, a high-frequency (25–80 Hz) power modulation was seen that was significant in 5/9 hippocampi tested (Figure 3). No changes in power under 20 Hz was observed at any recording site. If the theta-band activity is not power-modulated by eye movements, this indicates that either theta occurs independently of eye movements, or its phase is altered without a corresponding change in theta amplitude, as has been observed in other cognitive processes.

### Post-fixation phase alignment

**Figure 4:**
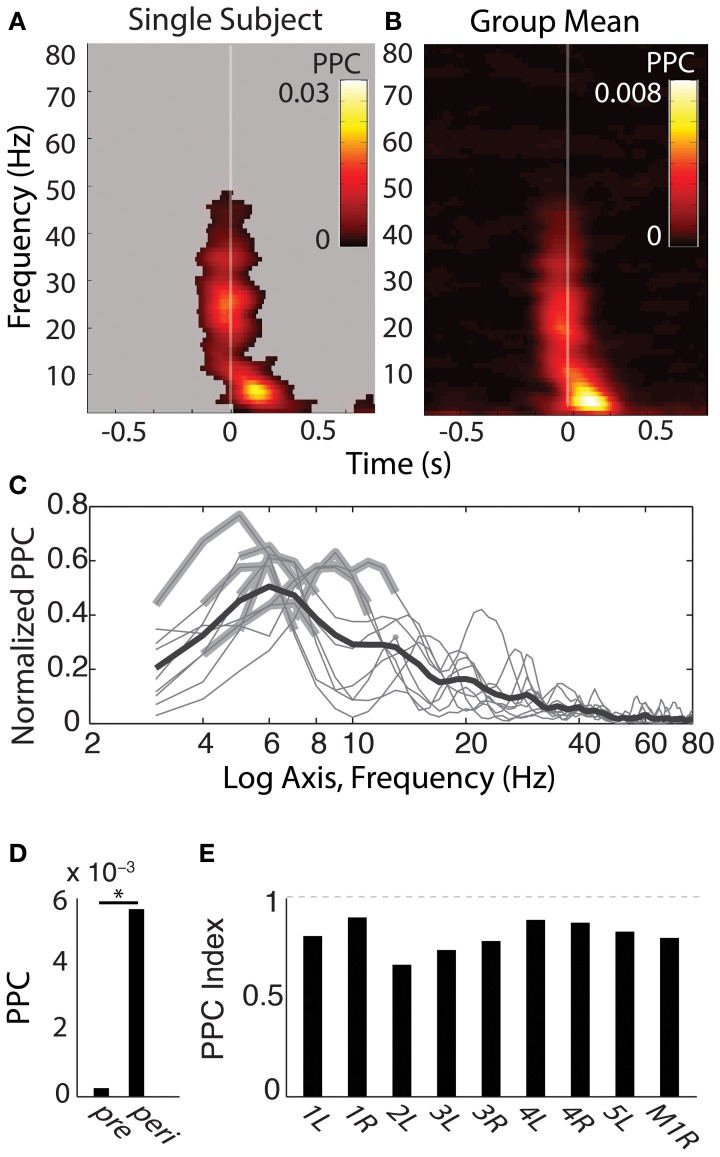
**Phase-alignment to fixations during search shows specificity in frequency and time. (A)** Single-subject theta-band phase-locking to fixations. Shown is the pairwise phase consistency (PPC) for each time-frequency bin described in Figure 3, with time on the x axis relative to fixation onset and frequency on the y-axis. Significant PPC values are unmasked (*p* < 0.001 of fixation-time-shuffled distribution and Rayleigh test *p* < 0.001 FDR corrected). **(B)** Group-averaged PPC plots for each hippocampus sampled. Conventions are as in **(A)**, but for the mean PPC values, with no masking. **(C)** Frequency band-limited hippocampal phase alignment to fixations, across subjects. For each frequency indicated on the x axis, the y-axis shows the mean PPC value from time-frequency points centered −50 to 250 ms from fixation onset, expressed as the proportion of the maximal PPC value of that sample from 3 to 80 Hz and −800 to 800 ms. Each thin line reflects the normalized PPC from a single hippocampus, with significant frequency bands indicated with a thickened gray line; all hippocampi produced significant phase concentration within a 3–8 Hz band; none produced significant phase concentration above 12 Hz. The black bold line is the group-averaged mean (*N* = 9). **(D)** Peri-fixational phase alignment, group averages pre and peri-fixation. Average PPC values from time bins centered −50 to 250 ms from fixation onset were compared with PPC values −800 to −600 ms prior to each fixation. ^*^p < 0.01, Wilcoxon signed rank test. **(E)** Bars reflect the PPC values from each hippocampus recorded, indexed as the difference between the pre- and peri-fixational time windows (see Materials and Methods). Participant number and hemisphere are indicated below each bar.

SEMs elicited phase alignment in all hippocampal recording sites from all participants tested (for example see Figure 4A, for group mean, see Figure 4B). PPC results were qualitatively indistinguishable to those obtained using the PLV measure, so only the PPC values are depicted. The alignment was typically seen within the first 200 ms following fixation, and was restricted to a band that peaked within the 3–8 Hz range (Figure 4C; note non-significant harmonics of individual peaks at 10–16 Hz). In addition, 5/9 hippocampal recordings showed a brief PPC closely locked to the saccade event ranging between 15–35 Hz (Figures 4A,B), but presumably due to its transience, it did not survive significance testing in the −50 to 250 ms centered peri-fixation tests (Figure 4C). Furthermore, the beta-band but not theta-band phase alignment was seen in other sites that had evoked responses to saccades at that time, such as those in the temporal pole (Figure 2).

**Figure 5:**
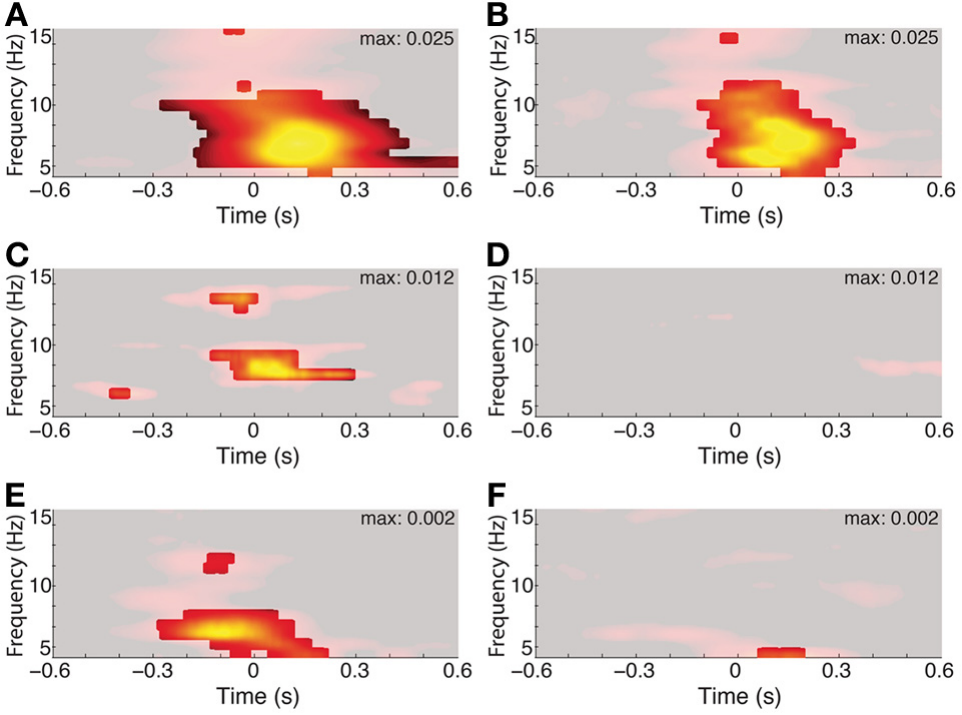
**Three to eight hertz phase alignment depends on visual search but not on matched-rate (3–6 Hz) saccades**. Left columns show full data and right columns the control condition. Significant PPC values are unmasked (*p* < 0.001 of fixation-time-shuffled distribution and Rayleigh test *p* < 0.001 FDR corrected). **(A)** PPC from a single hippocampal recording site in a patient. **(B)** PPC from the same site, excluding all but fixations lasting >500 ms both before and after the fixation onset of interest. **(C)** Average PPC from Patient 6 during search in the constant-stimulus version task. **(D)** Average PPC from the same sites, but for fixations made on the dark screen during the inter-trial intervals. **(E)** Average PPC from the macaque hippocampus aligned to fixations occurring during the main-task trials, and the inter-trial interval on a black screen **(F)**.

The duration of phase alignment and the frequency band overlapped with the average rate of saccades (2–6 Hz, Figure 1B). To determine whether hippocampal 3–8 Hz phase is passively reflecting the rate of eye movements, we calculated the PPC for the subset of fixations that were protracted in time (>500 ms fixations for both the aligned and the immediately preceding fixation, or <2 Hz). Figure 5A shows that even for this subset of long fixations, the 3–8 Hz phase effects seen from the full distribution persist. Phase alignment does not persist for longer than when shorter fixations are included, nevertheless, we see alignment at the same frequencies rather than decay or drop in frequency corresponding to the new saccade rate.

The effects of eye movements on hippocampal activity could be due to sampling new parts of the image, or they could be extra-retinal, reflecting some non-visual correlate of the eye movement itself. To address the relevance of the visual scene search to theta phase alignment, we compared the PPC during search to the PPC following fixations made on the darkened monitor, during the inter-trial intervals. Whereas all recording sites showed phase alignment within the 3–8 Hz band during scene search, no site showed this alignment from fixations that occurred during the ITI (Figures 5D,F).

Finally, the image alternation was occurring throughout visual search in the main task. To determine whether 3–8 Hz phase alignment occurred during visual search independent of the changing stimulus, we tested a patient on a constant-image control task. Theta phase alignment persisted during search on the static image, but was lost during the inter-trial interval, suggesting that image-guided visual search elicits the 3–8 Hz phase alignment.

## Discussion

Despite numerous differences across species, both human and monkey share a common mode of visual scene exploration. Capitalizing on the similarities in behavior, we conducted the same task in human and macaque and found a similar response in the hippocampus following fixations during visual search. The response, seen in all participants, was a 3–8 Hz frequency phase-alignment to the fixation. The effect was relatively short lived, lasting around or slightly longer than the typical duration of a fixation, amounting to 2 or 3 cycles of the oscillation. The effect was not driven solely by the rate of eye movements, because it persisted when only long-lasting <500 ms sequences of fixations were considered. The short time course even for long fixations suggests that the response is rapidly dampened, even without the interruption of a subsequent eye movement. Furthermore, the effect was not observed for eye movements made in between search trials, on a darkened monitor (Figure 5D), and even with a darkened environment (Figure 5F). Thus, the eye movement *per se* was not sufficient to elicit the observed phase-aligned activity, suggesting visual input and/or task-related factors contributed to the phase alignment. When a constant, unchanging scene was presented for the duration of search, the phase alignment persisted, suggesting that exogenous changes in visual input do not underlie the 3–8 Hz phase alignment (Figure 5C).

### Three to eight hertz phase alignment occurs without increases in power

Some of the earliest descriptions of theta phase resetting or aligning to an independent event, noted that it was not associated with increases in theta power (Buño et al., 1978; Givens, 1996). In the paper by Givens, a continuous conditional discrimination (CCD) showed phase-resetting where a sensory discrimination task did not. Theta power, however, was unchanged across tasks. In human ECoG recordings, theta phase-resetting was not associated with increases in power, suggesting a reorganization of oscillations rather than an evoked response (Rizzuto, 2003). During a Sternberg item recognition task, resetting was the predominant effect, and over a larger spatial extent. Another account of phase alignment without power changes was shown to result from sclerosis (Mormann, 2007), which would be relevant to the patient population; however, in the present study, individual hippocampi with no known or visible signs of pathology also showed phase-alignment in the absence of power changes (Figures 4C,D).

### Cellular correlates of theta resetting in the hippocampus that account for no change in power

The lack of power changes is consistent with spiking data (Vinogradova, 1995; Zugaro et al., 2005) in which theta phase reset is associated with no significant increase in spiking. On the contrary, a resetting stimulus first results in complete quiescence within the hippocampus, followed by periodic increases in spiking activity at the theta frequency. The maxima of the peaks, however, are not greater than the spike rates prior to the resetting stimulus. Thus, increases in spike-field coherence rather than overall firing rate are predicted for the post-saccadic activity. This would be consistent with human unit recordings during a visual recognition memory task in which theta phase-locking but not spike-triggered power distinguished later-remembered vs. later-forgotten trials (Rutishauser et al., 2010). Even in rodents known for sustained hippocampal theta, the spiking activity used to decode the spatiotemporal context occurs within one cycle and can, under some circumstances, flip as rapidly as one theta cycle, suggesting that the underlying coding can be accomplished within this time frame (Jezek et al., 2011).

### Phase clustering and power increases

A notable difference between our results and other intracranial results looking at hippocampal responses is that we see only phase effects whereas often both power and phase increases are identified. This suggests that the mechanisms are dissociable in the hippocampus and may be operative under different conditions. A detailed analysis was conducted of the hippocampal potentials during a continuous visual word recognition paradigm in which a button press indicated old or new judgments (Mormann et al., 2005). Phase locking occurred for both hits and correct rejections in the theta and alpha bands. Power increases were greater for hits than for correct rejections, thus, the phase locking they describe appears to be different from the evoked responses in this memory task. In the present study, the lack of phase alignment in the ITI, despite saccade generation, suggests that phase alignment is specific to post-saccadic (retinal) processing rather than an automatic result of the post-saccadic (or extra-retinal) state.

### Higher-frequency phase and power effects

For some participants, hippocampal power above 25 Hz was enhanced in a narrow window around the time of the saccade. In addition, phase alignment was seen in some individual sites at 15–35 Hz, also locked to the saccade event, but none survived the statistical testing in the peri-event window used here. These modulations—especially those also observed in the temporal pole—may be attributable to oculomotor artifact, even in intracranial recordings (Yuval-Greenberg et al., 2008; Kovach et al., 2011; Nagasawa et al., 2011); however, visually-modulated neural responses are also known to occur in early visual areas in these frequency bands (Ito et al., 2013), and gamma oscillations are thought to play an important role in hippocampal computation in rats (Colgin et al., 2009) and primates (Jutras et al., 2009), therefore, the higher frequency responses are not necessarily attributable to eye movement artifacts. In contrast to the higher-frequency responses, the protracted hippocampal response was of lower frequency than that described for oculomotor artifacts, did not modulate power, was not limited to the eye movement duration, showed a polarity reversal across two probes spanning the hippocampus mediolaterally, and was additionally task-dependent: there was greater phase alignment when eye movements were made during the search task than during the inter-trial interval, disambiguating it from other responses.

### Whether and which theta

The 3–8 Hz response in this task was among the most consistent and robust effects, with all participants showing this band in response to fixation. Like other studies with humans, macaques, and bats, clear theta rhythmicity in this study appears to be shorter-lived than what is seen in the rat. For eye movements, this may fall conveniently within the fixation durations, yet we did not observe a “ringing” or sustained theta commensurate with the sustained fixation windows. One possibility is that the nature of sampling strongly constrains the hippocampal oscillations; the greater the periodicity of movement, the more likely the hippocampus will entrain to it. Alternatively, hippocampal theta in primates may simply not have the same resonance properties as that of the rat hippocampus. Another non-exclusive possibility is that the hippocampus here is matching extra-hippocampal oscillations. Both of these possibilities are broadly supported by the short-lived (500 ms) theta coherence associated with successful memory encoding among hippocampal and other neocortical structures (Burke et al., 2013), theta synchronization associated with visual search (Bosman et al., 2010) and the long-range theta coherence in fronto-parietal networks during planning epochs that are initiated by eye fixations (Phillips et al., 2013). Finally, if eye movements trigger a band-limited response to a single event, the typical rates of repetition of eye movements could nevertheless, produce effects at the cellular level that may be indistinguishable from those produced through sustained or intrinsic rhythms. As such, eye movements could still co-opt the functionality associated with these rhythms as they have been observed in rats. In any case, the hippocampus of primates is sensitive to visual exploratory rhythms; how this constrains or facilitates hippocampal function remains to be seen.

### Conflict of interest statement

The authors declare that the research was conducted in the absence of any commercial or financial relationships that could be construed as a potential conflict of interest.
